# Relating Darcy-Scale Chemical Reaction Order to Pore-Scale Spatial Heterogeneity

**DOI:** 10.1007/s11242-022-01817-0

**Published:** 2022-07-15

**Authors:** Po-Wei Huang, Bernd Flemisch, Chao-Zhong Qin, Martin O. Saar, Anozie Ebigbo

**Affiliations:** 1 Geothermal Energy and Geofluids Group, Institute of Geophysics, Department of Earth Sciences, ETH Zurich, Zurich, Switzerland; 2 Institute for Modelling Hydraulic and Environmental Systems, University of Stuttgart, Stuttgart, Germany; 3 State Key Laboratory of Coal Mine Disaster Dynamics and Control, Chongqing University, Chongqing, China; 4 Department of Earth and Environmental Sciences, University of Minnesota, Minneapolis, USA; 5 Chair of Hydromechanics, Helmut Schmidt University, Hamburg, Germany

**Keywords:** Reactive transport, Mineral dissolution, Upscaling, Reaction rate law

## Abstract

Due to spatial scaling effects, there is a discrepancy in mineral dissolution rates measured at different spatial scales. Many reasons for this spatial scaling effect can be given. We investigate one such reason, i.e., how pore-scale spatial heterogeneity in porous media affects overall mineral dissolution rates. Using the bundle-of-tubes model as an analogy for porous media, we show that the Darcy-scale reaction order increases as the statistical similarity between the pore sizes and the effective-surface-area ratio of the porous sample decreases. The analytical results quantify mineral spatial heterogeneity using the Darcy-scale reaction order and give a mechanistic explanation to the usage of reaction order in Darcy-scale modeling. The relation is used as a constitutive relation of reactive transport at the Darcy scale. We test the constitutive relation by simulating flow-through experiments. The proposed constitutive relation is able to model the solute breakthrough curve of the simulations. Our results imply that we can infer mineral spatial heterogeneity of a porous media using measured solute concentration over time in a flow-through dissolution experiment.

## Introduction

Geochemical reactions such as mineral dissolution play an essential role in determining water chemistry, soil formation, biogeochemical cycling, and global climate (Wen and Li 2017). Mineral reactions can also occur by injecting CO$_{2}$ in subsurface reservoirs during geothermal energy extraction (Randolph and Saar 2011; Adams et al. 2021; Ezekiel et al. 2022) or calcite mineralization (Pogge von Strandmann et al. 1983). One of the most significant obstacles to understanding the geochemical reactivity of natural subsurface environments stems from the multitude of spatial scales that have to be considered (Noiriel et al. 2012). Due to spatial scaling effects, mineral dissolution rates are known to be 3–6 orders of magnitude lower in the field than when measured in the laboratory (White and Brantley 2003; Navarre-Sitchler and Brantley 2007; Maher 2010; Moore et al. 2012). The deviation in mineral dissolution rates strongly limits the extrapolation of kinetic dissolution models and parameters characterized at the laboratory to natural systems (Li et al. 2020).

Many factors are responsible for the spatial scaling effects of mineral dissolution rates. This work focuses on how pore-size distribution—which contributes to hydraulic heterogeneity—and spatial mineral distribution causes such spatial scaling effects. Li et al. (2007) performed simulations with various settings of spatial mineral distribution and concluded that spatial mineral distribution has a significant scaling effect when the reactive minerals are of small but typical proportions. Experimental studies using a column packed with quartz and magnesite have confirmed the significant role of spatial heterogeneities in subsurface reactive transport and can be used to quantify the effect of spatial mineral distribution on dissolution rates (Salehikhoo et al. 2013; Li et al. 2014; Li and Salehikhoo 2015). X-ray micro-tomography provides observations of the impact of physical and chemical heterogeneity on reaction rates in multimineral porous media (Tutolo et al. 2015; Luhmann et al. 2017; Al-Khulaifi et al. 2017, 2018, 2019; Menke et al. 2016, 2018). Fischer et al. (2014) and Fischer and Luttge (2017) studied how mineral surface roughness at the nanometer scale affects surface reaction rates and proposed to upscale the mineral reaction rate using Monte Carlo simulations. Ma et al. (2019, 2021) quantified the accessible surface area of minerals in a sandstone using scanning electron microscopy (SEM) images and Brunauer–Emmett–Teller (BET) surface area measurements.

Regarding the influence of hydraulic heterogeneity, Wen and Li (2017) and Jung and Navarre-Sitchler (2018a) performed reactive transport simulations on stochastically generated permeability fields and studied how hydrologic heterogeneity affects mineral dissolution rates. Using Monte Carlo simulations, Jung and Navarre-Sitchler (2018b) further studied the time dependency of mineral dissolution rates, and Wen and Li (2018) developed an upscaled rate law for mineral dissolution in heterogeneous media under variable residence-time and length-scale conditions. Li et al. (2020) upscaled mineral dissolution rates in a porous medium with a random permeability field using the fluid travel-time distribution function. The works mentioned above used the transition state theory (Lasaga 1998) to model mineral dissolution rates, with a macroscale reaction order of unity $(n=1)$. This is reasonable since there are no physical explanations why this macroscale reaction order should not be one (Lasaga 1998; Brantley and Conrad 2008).

However, there are rate models with a macroscale reaction order of 2 in kinetics of crystal growth (Nancollas 1968; Reddy 1975, 1977). Such second-order kinetics are used for modeling surface spiral growth (Nielsen 1984). Considering calcite as our mineral of particular interest, fitting experimental data using a reaction order larger than 1 is common, especially when the saturation is close to equilibrium (Plummer and Wigley 1976; Plummer et al. 1978; Palmer 1991; Svensson and Dreybrodt 1992). A higher reaction order is also observed in modeling calcite dissolution in seawater (e.g., Subhas et al. 2015; Naviaux et al. 2019).

In this work, we use analytical techniques to develop a constitutive relation of mineral dissolution kinetics in porous media based on models with a reaction order ${n>1}$. We characterize hydraulic heterogeneity and mineral spatial heterogeneity by the longitudinal dispersivity and the reaction order. We use the bundle-of-tubes analogy to show how the reaction order relates to both hydraulic and spatial mineral heterogeneity in porous media. Furthermore, we simulate experimental scenarios involving advective and dispersive transport using such a constitutive relation. Our results show how concentration breakthrough curves of the reactive species reveal information of both the hydraulic and chemical heterogeneity of porous media.

## Materials and Methods

This section first introduces reactive transport models at the pore scale and the Darcy scale. Then, we lay out statistical distributions of pore sizes and effective-surface-area ratios. Such distributions can thus define the volume-averaged concentration. Constitutive relations based on the Darcy-scale reaction order is established using Taylor series expansions. We check the applicability of the constitutive relations by comparing the modeled concentration and the volume-averaged concentration using a goodness-of-fit measure, the Jensen–Shannon divergence. Finally, we explain how one can apply the proposed constitutive relation using a flow-through experiment.

### Reactive Transport at the Pore Scale

At the pore scale, we model the transport of a mineral-forming solute by

1 $$\begin{aligned} \frac{\partial C^{*}}{\partial t^{*}} + \vec {u} \cdot \nabla C^{*} - \nabla \cdot ( D \nabla C^{*}) = 0, \end{aligned}$$

where $C^{*}$ is the solute concentration in the fluid (mol m$^{-3}$), $\vec {u}$ is the fluid velocity (m s$^{-1}$), *D* is the molecular diffusivity (m$^{2}$ s$^{-1}$). In Eq. (1), we consider $C^{*}$ as the unknown variable, and the fluid velocity, $\vec {u}$, is given. The heterogeneous reaction is defined as

2 $$\begin{aligned} {\hat{{\varvec{n}}}} \cdot D \nabla C^{*} = R_{\mathrm{het}}(C^{*}), \end{aligned}$$

where ${\hat{{\varvec{n}}}}$ is the unit normal vector pointing outwards from the fluid to the solid surface, and $R_{\mathrm{het}}$ is the rate of the heterogeneous reaction per surface area (mol m$^{-2}$ s$^{-1}$) as a function of $C^{*}$. Since we consider a bundle of capillary tubes as a model of a porous medium, we introduce Eq. (1) described in cylindrical coordinates:

3 $$\begin{aligned} \frac{\partial C^{*}}{\partial t^{*}} + u_{z} \frac{\partial C^{*}}{\partial z^{*}} - D \left[ \frac{1}{r^{*}} \frac{\partial }{\partial r^{*}} \left( r^{*} \frac{\partial C^{*}}{\partial r^{*}} \right) + \frac{\partial ^{2} C^{*}}{\partial z^{*2} } \right] = 0, \end{aligned}$$

and the boundary condition, Eq. (2),

4 $$\begin{aligned} D \frac{\partial C^{*}}{\partial r^{*}} = R_{\mathrm{het}}(C^{*}) \qquad {\text{at }} r^{*} = r, \end{aligned}$$

where *r* is the radius of the capillary tube (m). We assume fully developed axisymmetric fluid flow, $u_{r}=u_\theta =0$, constant molecular diffusivity, and axisymmetric solute concentration. Furthermore, we assume the heterogeneous reaction causes negligible change to the tube radius and the mineral surface area. Regarding dissolution and precipitation reactions leading to changes in the pore geometry of porous media and fractures, we refer the reader to the works of Sallés et al. (1993), Békri et al. (1995, 1997) for further discussion. Following Arce et al. (2005), the area-averaged concentration of the capillary tube is

5 $$\begin{aligned} \langle C^{*} \rangle = \frac{1}{\pi r^{2}} \int _{0}^{r} 2 \pi r^{*} C^{*} {\mathrm{d}}r^{*} = \frac{2}{r^{2}} \int _{0}^{r} r^{*} C^{*} {\mathrm{d}}r^{*}. \end{aligned}$$

To develop an area-averaged or upscaled equation, we perform area-averaging of Eq. (3) to obtain

6 $$\begin{aligned} \frac{\partial \langle C^{*} \rangle }{\partial t^{*}} + \frac{\partial \langle u_{z} C^{*} \rangle }{\partial z^{*}} - \frac{2 D}{r^{2}} \int _{0}^{r} \frac{\partial }{\partial r^{*}} \left( r^{*} \frac{\partial C^{*}}{\partial r^{*}} \right) {\mathrm{d}}r^{*} - D \frac{\partial ^{2} \langle C^{*} \rangle }{\partial z^{*2} } = 0. \end{aligned}$$

Evaluate the integral and rearrange:

7 $$\begin{aligned} \frac{\partial \langle C^{*} \rangle }{\partial t^{*}} + \frac{\partial \langle u_{z} C^{*} \rangle }{\partial z^{*}} - D \frac{\partial ^{2} \langle C^{*} \rangle }{\partial z^{*2} } = \frac{2 D}{r} \frac{\partial C^{*}}{\partial r^{*}} \bigg |_{r^{*}=r}. \end{aligned}$$

Following Paine et al. (1983), we further expand the convective term using Gray (1975)’s representation:

8 $$\begin{aligned} \frac{\partial \langle u_{z} C^{*} \rangle }{\partial z^{*}} = \underbrace{\langle u_{z} \rangle \frac{\partial \langle C^{*} \rangle }{\partial z^{*}}}_{\text{convective transport}} + \underbrace{\frac{\partial \langle {\tilde{u}}_{z} {\widetilde{C}}^{*} \rangle }{\partial z^{*}}}_{\text{dispersive transport}}, \end{aligned}$$

where ${\tilde{u}}_{z}$ and ${\widetilde{C}}^{*}$ are the spatial deviation terms of the fluid velocity and solute concentration, respectively. Considering only transport, Paine et al. (1983) showed that the dispersive transport term can be represented by the Taylor–Aris theory of dispersion:

9 $$\begin{aligned} \frac{\partial \langle {\tilde{u}}_{z} {\widetilde{C}}^{*} \rangle }{\partial z^{*}} = - \frac{\langle u_{z} \rangle ^{2} r^{2}}{48 D} \frac{\partial ^{2} \langle C^{*} \rangle }{\partial z^{*2} } \end{aligned}$$

with the constraints $\langle u_{z} \rangle r/D \gg 1$ and $D t^{*} /r^{2} \gg 1$. Ananthakrishnan et al. (1965) delineated dispersion effects for $\langle u_{z} \rangle r/D = O(1)$ and introduced a time-dependent dispersion coefficient (m$^{2}$ s$^{-1}$) to model dispersion in the regime not covered by the Taylor–Aris dispersion. We utilize the time-dependent dispersion coefficient obtained by the method of moments (Barton 1983) and asymptotic techniques (Vrentas and Vrentas 1988)

10 $$\begin{aligned} {\mathscr{D}}_{{\mathrm{L}}} = \frac{\langle u_{z} \rangle ^{2} r^{2}}{48 D} - \frac{64 \langle u_{z} \rangle ^{2} r^{2}}{D} \sum _{n=1}^{\infty } \frac{e^{-j_{n}^{2} D t^{*}/r^{2}}}{j_{n}^{6}}, \end{aligned}$$

where $j_{n}$ is the *n*th root of Bessel function of the first kind of order 1 (Meng and Yang 2017). The time-dependent term in ${\mathscr{D}}_{{\mathrm{L}}}$ serves as a correction term for Taylor–Aris dispersion. Such a definition of dispersive transport is suitable for simple initial conditions, e.g., injecting a pulse of solute. Considering general initial conditions under steady flow conditions, one has to include source terms to address the complexity of dispersive transport in a cylindrical tube (Taghizadeh et al. 2020). Since the averaged convection term can be described by averaged quantities, we divert our focus to the source term on the right-hand side of Eq. (7):

11 $$\begin{aligned} \frac{2 D}{r} \frac{\partial C^{*}}{\partial r^{*}} \bigg |_{r^{*}=r} = \frac{2}{r} R_{\mathrm{het}}(C^{*}|_{r^{*}=r}). 
\end{aligned}$$

The heterogeneous reaction term depends on the solute concentration at the solid–fluid boundary in the radial direction. For reaction–diffusion systems in a cylindrical tube with a linear irreversible heterogeneous reaction, ${R_{\mathrm{het}}=-k C^{*} |_{r^{*}=r}}$, Arce et al. (2005) performed an order-of-magnitude estimate and claimed that when $kr/D \ll 1$, the solute concentration on the boundary can be approximated by

12 $$\begin{aligned} C^{*}|_{r^{*}=r} = \langle C^{*} \rangle , \end{aligned}$$

where *k* is a rate constant (m s$^{-1}$). The constraint $kr/D \ll 1$ gives us a good range of *k*, since the pore radius is usually some tens of micrometers. Combining Eqs. (7)–(12), the area-averaged solute transport equation in a cylindrical pore reads

13 $$\begin{aligned} \frac{\partial \langle C^{*} \rangle }{\partial t^{*}} + \langle u_{z} \rangle \frac{\partial \langle C^{*} \rangle }{\partial z^{*}} - \left( D + {\mathscr{D}}_{\mathrm{L}} \right) \frac{\partial ^{2} \langle C^{*} \rangle }{\partial z^{*2} } = \frac{2}{r} \langle R_{\mathrm{het}} \rangle (\langle C^{*} \rangle ). \end{aligned}$$

This is an ad hoc approach of developing the averaged model. Though not perfect, such a one-dimensional (1D) expression does represent reactive transport in a pore throat in many pore-network models (Algive et al. 2010; Raoof et al. 2012, 2013; Varloteaux et al. 2013a, b; Qin and Hassanizadeh 2015; Bekri et al. 2015; Gostick et al. 2016; Xiong et al. 2016; Esteves et al. 2020). Rigorous upscaling of reactive flow in thin geometries has been performed with the following considerations: general mass action kinetics (van Duijn and Pop 2004), dominant Péclet and Damköhler numbers (Mikelić et al. 2006; van Duijn et al. 2008), changes in pore-scale geometry (van Noorden 2009b; Kumar et al. 2011), changes in pore-scale geometry with non-isothermal effects (Bringedal et al. 2015, 2016), changes in pore-scale geometry with two-phase flow (von Wolff and Pop 2021), and coexisting homogeneous reactions (Boso and Battiato 2013). For perforated porous media, rigorous upscaling of reactive flow involving dissolution or precipitation processes has been performed (Kumar et al. 2016), with considerations of changing pore-scale geometry (van Noorden 2009a) as well as multiphase reaction–diffusion systems (Redeker et al. 2016).

The dimensionless number, *kr*/*D*, is often identified as the pore-scale Thiele modulus squared $\phi ^{2}$ or the Damköhler number Da. Balakotaiah et al. (1995) used the invariant manifold expansion for advection and heterogeneous reaction in a cylindrical tube and showed that when $\phi ^{2} \ll 1$, dispersion effects can be modeled by the Taylor–Aris theory. For reaction–diffusion systems in porous media, Valdés-Parada et al. (2017) showed that the constraint $\phi ^{2} \ll 1$ can be loosened to $\phi ^{2} \le 1$ by modifying the effective diffusivity and the effective reaction rate constant at the macro scale, and Bourbatache et al. (2020) recovered classical homogenized diffusive equations for small values of Damköhler numbers (defined by the length scale of the representative elementary volume).

#### Second-Order Dissolution Kinetics

Throughout this work, the heterogeneous reaction of interest is mineral dissolution. A common mineral dissolution model is of second order:

14 $$\begin{aligned} R_{\mathrm{het}} = k_{\mathrm{d}} - k_{\mathrm{p}}^{\mathrm{II}} C^{*2}, \end{aligned}$$

where $k_{\mathrm{d}}$ is the dissolution rate constant (mol m$^{-2}$ s$^{-1}$) and $\smash [b]{k_{\mathrm{p}}^{\mathrm{II}}}$ is the precipitation rate constant of second-order kinetics (mol$^{-1}$ m$^{4}$ s$^{-1}$). We assume the solution is dilute, such that the activity coefficient of the solute is unity. We relate the dissolution model with a more prevalent formulation involving the solubility product constant, $K_{\mathrm{sp}}$, and the ion activity product, IAP:

15 $$\begin{aligned} R_{\mathrm{het}} = k_{\mathrm{d}} \left( 1-\frac{\mathrm{IAP}}{K_{\mathrm{sp}}}\right) \, . \end{aligned}$$

One can switch between the two forms by stating $\mathrm{IAP}=C^{*2}$ and $K_{\mathrm{sp}}=k_{\mathrm{d}}/k_{\mathrm{p}}^{\mathrm{II}}$. Using calcite as an example, when pH$>5.5$, the dominant dissolving species at the diffusion boundary layer are Ca$^{2+}$ and CO$_{3}^{2-}$ (Sjöberg and Rickard 1984). Thus, at intermediate pH values, $\mathrm{IAP} = C^{*}_{{\mathrm{Ca}}^{2+}} C^{*}_{{\mathrm{CO}}_{3}^{2-}}$. The assumption of electroneutrality close to the mineral surface yields $\mathrm{IAP} = (C^{*}_{{\mathrm{Ca}}^{2+}})^{2}$, which results in second-order dissolution kinetics (Ebigbo et al. 2012; Levenson and Emmanuel 2013). For magnesite dissolution at neutral to alkaline pH regimes, such second-order kinetics is also suitable (Salehikhoo et al. 2013; Wen and Li 2017). Second-order dissolution/precipitation kinetics also appears in upscaling reactive transport processes in porous media with attention to moving solid–fluid interfaces (Ray et al. 2019; Bringedal et al. 2020; Gärttner et al. 2020). Combining Eqs. (13) and (14) yields

16 $$\begin{aligned} \frac{\partial \langle C^{*} \rangle }{\partial t^{*}} + \langle u_{z} \rangle \frac{\partial \langle C^{*} \rangle }{\partial z^{*}} - \left( D + {\mathscr{D}}_{\mathrm{L}} \right) \frac{\partial ^{2} \langle C^{*} \rangle }{\partial z^{*2} } = \frac{2}{r} \left( k_{\mathrm{d}} - k_{\mathrm{p}}^{\mathrm{II}} \langle C^{*2} \rangle \right) . \end{aligned}$$

We relate the dimensional and nondimensional quantities by

17 $$\begin{aligned} t^{*} = [t]\,t,\,z^{*} = L_{z} \, z,\,\langle C^{*} \rangle = [C]\,C, \end{aligned}$$

where *t* is the nondimensional time, *z* is the nondimensional space, *C* is the nondimensional solute concentration, and $L_{z}$ is the length of the cylindrical pore (m). Then, we nondimensionalize Eq. (16) by the following scaling of time and concentration

18 $$\begin{aligned}{}[t]=\frac{L_{z}^{2}}{D}, \quad [C]=\sqrt{\frac{k_{\mathrm{d}}}{\smash [b]{k_{\mathrm{p}}^{\mathrm{II}}}}}, \end{aligned}$$

where [*t*] is the diffusive time scale (s). The variables in square brackets remove the physical dimension of the starred variables and refer to characteristic quantities. Thus we have a nondimensional equation of reactive transport

19 $$\begin{aligned}&\frac{\partial C}{\partial t} + {\text{Pe}} \frac{\partial C}{\partial z} - \left( 1 + \frac{ ({\text{Pe}} \,\epsilon )^{2}}{48} - 64 ({\text{Pe}}\, \epsilon )^{2} \sum _{n=1}^{\infty } \frac{e^{-j_{n}^{2} \epsilon ^{-2} t}}{j_{n}^{6}} \right) \frac{\partial ^{2} C}{\partial z^{2}} = {\text{Da}}(1-C^{2}), \end{aligned}$$

20 $$\begin{aligned}&\epsilon = \frac{r}{L_{z}}, \quad {\text{Pe}} = \frac{\langle u_{z} \rangle L_{z}}{D}, \quad {\text{Da}} = \frac{2}{r} \frac{L_{z}^{2} \sqrt{k_{\mathrm{d}} \smash [b]{k^{\mathrm{II}}_{\mathrm{p}}}}}{D} = \frac{2}{\epsilon ^{2}} \frac{ r\sqrt{k_{\mathrm{d}} \smash [b]{k^{\mathrm{II}}_{\mathrm{p}}}} }{D}, \end{aligned}$$

where $\epsilon$ is the aspect ratio, Pe is the Péclet number, and Da is the Damköhler number. In Eq. (20), we further relate Da to $\epsilon$ and the pore-scale Thiele modulus (of second-order reactions), denoted as $r\sqrt{k_{\mathrm{d}} \smash [b]{k^{\mathrm{II}}_{\mathrm{p}}}}/D$, to show that for such a definition of the Damköhler number, ${\text{Da}} > 1$ does not necessarily break the assumption of a small Thiele modulus. For a slender cylindrical pore, $\epsilon \ll 1$, and a controlled flow rate, ${\text{Pe}}\, \epsilon \ll 1$, we neglect the effect of dispersion. The $\exp \left( {-j_{n}^{2}\epsilon ^{-2}\, t}\right)$ term in the dispersion correction term also indicates such a correction term vanishes rapidly over time. Hence, the nondimensional reactive transport equation reads

21 $$\begin{aligned} \frac{\partial C}{\partial t} + {\text{Pe}} \frac{\partial C}{\partial z} - \frac{\partial ^{2} C}{\partial z^{2}} = {\text{Da}}(1-C^{2}). \end{aligned}$$

#### First-Order Dissolution Kinetics

First-order-kinetic models are uncommon, since chemical reactions often involve two reagents (Cussler 2009). If one wanted to make use of first-order kinetics, one would have to assume the concentration of a mineral-forming ion is in excess or constant (Meile and Tuncay 2006) or limit the usage of first-order kinetics to low solute concentrations (Kaufmann and Dreybrodt 2007). Nonetheless, we introduce the model of first-order mineral dissolution

22 $$\begin{aligned} R_{\mathrm{het}} = k_{\mathrm{d}} - k_{\mathrm{p}}^{\mathrm{I}} C^{*}, \end{aligned}$$

where $k_{\mathrm{p}}^{\mathrm{I}}$ is the precipitation-rate constant of first-order kinetics (m s$^{-1}$). First-order dissolution kinetics is utilized to model evolution of karst aquifers (Gabrovšek and Dreybrodt 2001). They apply to dissolution rates of various minerals such as gypsum, rocksalt, calcium carbonate, and quartz (Jeschke and Dreybrodt 2002). Considering a general surface reaction, first-order kinetic models resemble adsorption and desorption kinetics studied by, e.g., Zhang et al. (2017), as well as the Noyes–Whitney model of drug dissolution (Dokoumetzidis and Macheras 2006). First-order kinetics is also applied to heterogeneous reactions between living cells and extracellular fluids (Santos-Sánchez et al. 2016). Combining Eqs. (13) and (22) and ignoring dispersion effects, we scale time and concentration by

23 $$\begin{aligned}{}[t]=\frac{L_{z}^{2}}{D}, \quad [C]=\frac{k_{\mathrm{d}}}{k_{\mathrm{p}}^{\mathrm{I}}}, \end{aligned}$$

such that the Péclet and the Damköhler numbers are

24 $$\begin{aligned} {\text{Pe}} = \frac{\langle u_{z} \rangle L_{z}}{D}, \quad {\text{Da}} = \frac{2}{r} \frac{L_{z}^{2} k_{\mathrm{p}}^{\mathrm{I}}}{D}\,=\frac{2}{\epsilon ^{2}} \frac{ k_{\mathrm{p}}^{\mathrm{I}} r}{D}. \end{aligned}$$

Notice the pore-scale Thiele modulus squared, $\phi ^{2} = kr/D$, appears in the form of the precipitation-rate constant, $k_ {\mathrm{p}}^{\mathrm{I}}$. Since $\phi ^{2}$ is proportional to ${\text{Da}} \, \epsilon ^{2}$, the assumption of $\phi ^{2} \ll 1$ is fulfilled, similar to the relation between the Damköhler number and the pore-scale Thiele modulus of second-order kinetics. The nondimensional reactive transport equation of first-order kinetics is therefore

25 $$\begin{aligned} \frac{\partial C}{\partial t} + {\text{Pe}} \frac{\partial C}{\partial z} - \frac{\partial ^{2} C}{\partial z^{2}} = {\text{Da}}(1-C). \end{aligned}$$

As for other possible models of heterogeneous kinetics, Qiu et al. (2017) provided a comprehensive review of upscaling reactive transport processes in porous media, e.g., Michaelis–Menten-type kinetics (Wood et al. 2007; Dadvar and Sahimi 2007), Monod-type kinetics (Heße et al. 2009), nonlinear kinetics that reduce to first-order kinetics when the reaction order is 1 (Guo et al. 2015).

We define the average velocity in a cylindrical pore using the Hagen–Poiseuille equation

26 $$\begin{aligned} \langle u_{z} \rangle = \frac{r^{2}}{8} \frac{\Delta P}{\eta L_{z}}, \end{aligned}$$

where $\eta$ is the dynamic viscosity of the fluid (Pa s) and $\Delta P$ is the pressure difference between the inlet and the outlet (Pa).

### Reactive Transport at the Darcy Scale

We use the bundle-of-tubes analogy to model reactive transport at the Darcy scale (Kozeny 1927). We define the specific mineral surface area (m$^{-1}$) of the porous medium as

27 $$\begin{aligned} S = \frac{A}{V} = \frac{2 L_{z} \pi \sum _{i=1}^{N} \omega _{i} r_{i}}{V}, \end{aligned}$$

where *A* is the mineral surface area, *V* is the bulk volume of the porous media (m$^{3}$), $\omega$ is the ratio between mineral surface area and the total surface area of a pore, and *N* is the total number of pores. We assume that the pores have the same length, $L_{z}$, as the porous medium, such that the tortuosity is 1. The porosity of the porous medium is

28 $$\begin{aligned} \psi = \frac{V_{\mathrm{f}}}{V} = \frac{L_{z} \pi \sum _{i=1}^{N} (r_{i})^{2}}{V}, \end{aligned}$$

where $V_{\mathrm{f}}$ is the fluid volume. Dividing Eq. (27) by Eq. (28), we obtain

29 $$\begin{aligned} \frac{S}{\psi } = \frac{A}{V_{\mathrm{f}}} = \frac{2 \sum _{i=1}^{N} \omega _{i} r_{i}}{\sum _{i=1}^{N} (r_{i})^{2}}. \end{aligned}$$

Recall the Damköhler number for first-order kinetics, Eq. (24), the Damköhler number at the Darcy scale is therefore

30 $$\begin{aligned} {\text{Da}}_{\mathrm{d}} = \frac{S}{\psi } \frac{L_{z}^{2} k_{\mathrm{p}}^{\mathrm{I}}}{D}. \end{aligned}$$

where the characteristic length *L* is chosen to be the length of the porous medium, $L_{z}$. The Darcy-scale Damköhler number for second-order kinetics is

31 $$\begin{aligned} {\text{Da}}_{\mathrm{d}} = \frac{S}{\psi } \frac{L_{z}^{2} \sqrt{k_{\mathrm{d}} \smash [b]{k_{\mathrm{p}}^{\mathrm{II}}}} }{D}. \end{aligned}$$

Since we consider the porous medium as a bundle of tubes, the seepage velocity of the porous medium can be defined using a volume-averaged velocity,

32 $$\begin{aligned} \bar{u} = \frac{\sum _{i=1}^{N} \langle u_{i} \rangle (r_{i})^{2}}{\sum _{i=1}^{N} (r_{i})^{2}} = \frac{\sum _{i=1}^{N} (r_{i})^{4}}{8 \sum _{i=1}^{N} (r_{i})^{2}} \frac{\Delta P}{\eta L_{z}}. \end{aligned}$$

Hence, the Péclet number at the Darcy scale is

33 $$\begin{aligned} {\text{Pe}}_{\mathrm{d}} = \frac{\bar{u}L_{z}}{D}. \end{aligned}$$

Dispersion effects arise when the pore sizes are not uniform (Carbonell 1979; Arriaza and Ghezzehei 2013; Meng and Yang 2017). Therefore, we introduce a longitudinal dispersion coefficient (m$^{2}$ s$^{-1}$),

34 $$\begin{aligned} D_{\mathrm{L}} = \alpha _{\mathrm{L}} \bar{u}, \end{aligned}$$

where $\alpha _{\mathrm{L}}$ is the longitudinal dispersivity with the unit of length (m). We derive the longitudinal dispersion coefficient using the spatial moments of the averaged solute concentration of the bundle of tubes model

35 $$\begin{aligned} D_{\mathrm{L}} = \bar{u}^{2} t\, \left( \frac{\sum _{i=1}^{N} (r_{i})^{6} \sum _{i=1}^{N} (r_{i})^{2}}{\left( \sum _{i=1}^{N} (r_{i})^{4} \right) ^{2}} - 1 \right) = \bar{u}^{2} t\,f(r). \end{aligned}$$

The shape factor *f*(*r*) abbreviates the expression of pore sizes in Eq. (35). Detailed derivations are presented in “Appendix 1”. The longitudinal dispersivity is

36 $$\begin{aligned} \alpha _{\mathrm{L}} = \bar{u} t\,f(r) = \bar{z}\,f(r), \end{aligned}$$

where $\bar{z}$ is the center of mass of an instantaneous source injected in the porous domain. The spatial moment analysis showed that the longitudinal dispersivity increases linearly as the distance traveled of an instantaneous source, which is reasonable in an infinite domain. In recent studies based on pore-network modeling, Mahmoodlu et al. (2020) observed the longitudinal dispersivity increases as travel distance increases.

By scaling the longitudinal dispersion coefficient by the molecular diffusivity yields the nondimensional reactive transport model at the Darcy scale,

37 $$\begin{aligned} \frac{\partial C}{\partial t} + {\text{Pe}}_{\mathrm{d}} \frac{\partial C}{\partial z} - \left( 1 + ({\text{Pe}}_{\mathrm{d}})^{2} t\, f(r) \right) \frac{\partial ^{2} C}{\partial z^{2}} = q_{\mathrm{d}}, \end{aligned}$$

where $q_{\mathrm{d}}$ is the reaction term that is yet to be defined. Such a Darcy-scale model resembles the upscaled formulation of heterogeneous reaction systems including convective transport in porous media, which incorporates the effect of pore geometry in the dispersivity tensor and the effective reaction-rate constant (Wood et al. 2007; Battiato et al. 2009; Battiato and Tartakovsky 2011; Valdés-Parada et al. 2011; Guo et al. 2015; Qiu et al. 2017). Our proposed model assumes the reaction term does not contribute to the overall dispersion coefficient. The flow-through experiment introduced in Sect. 2.7 circumvents such effects by eliminating spatial variation of solute concentration. Note that when all pores have the same radius and effective-surface-area ratio, Eq. (37) reduces to the equation for a single pore. In the next section, we discuss statistical distributions of pore sizes and effective-surface-area ratios.

### Statistical Distributions of Pore Sizes and Effective-Surface-Area Ratios

The pore sizes of porous media usually follow a log-normal distribution (Shi et al. 1991; Hefny et al. 2020). The probability density function of log-normally distributed pore sizes, *r*, is

38 $$\begin{aligned} p(r) = \frac{1}{\sigma r \sqrt{2 \pi }}\exp { \left( - \frac{ (\log {(r)}-\mu )^{2} }{2 \sigma ^{2}} \right) }, \end{aligned}$$

where $\mu$ and $\sigma$ are the mean and standard deviation of the variable’s natural logarithm, respectively. The sum of pore sizes raised to the power of *m* can be described by a raw statistical moment,

39 $$\begin{aligned} \frac{1}{N} \sum _{i=1}^{N} (r_{i})^{m} = {{\text{E}}}(r^{m}) = \int _{0}^{\infty } r^{m} p(r) {\mathrm{d}}r. \end{aligned}$$

If the pore-size distribution is known, the intrinsic permeability, $k_{\mathrm{I}}$, of the bundle can be calculated as

40 $$\begin{aligned} k_{\mathrm{I}} = \frac{\sum _{i=1}^{N} (r_{i})^{4}}{8 \sum _{i=1}^{N} (r_{i})^{2}} = \frac{{{\text{E}}}(r^{4})}{8 {{\text{E}}}(r^{2})}. \end{aligned}$$

Using the moments of the lognormal distribution, we can now clarify the shape factor,

41 $$\begin{aligned} f(r) = \exp {(4\sigma ^{2})}-1. \end{aligned}$$

Since we are interested in not only the pore-size distribution but also the effective surface area of the reactive minerals, we utilize the effective-surface-area ratio, $\omega$, which has a value between zero and one. A convenient choice for modeling the distribution of a variable bounded by zero and one is the beta distribution. The probability density function of the beta distribution is

42 $$\begin{aligned} p(\omega ) = \frac{\Gamma (a+b) \omega ^{a-1} (1-\omega )^{b-1}}{\Gamma (a) \Gamma (b)}, \end{aligned}$$

where $\Gamma (\cdot )$ is the gamma function. Variables *a* and *b* shape the beta distribution.

The specific surface area over porosity, $S/\psi$, which is a part of the Darcy-scale Damköhler number, Eqs. (30) and (31), can be defined as

43 $$\begin{aligned} \frac{S}{\psi } = \frac{2 \sum _{i=1}^{N} \omega _{i} r_{i}}{\sum _{i=1}^{N} (r_{i})^{2}} = \frac{2 {{\text{E}}}(\omega r) }{{{\text{E}}}(r^{2})}. \end{aligned}$$

Such a definition of $S/\psi$ depends on the pore geometry. Inferring from Hussaini and Dvorkin (2021)’s compilation of specific surface area versus porosity using digital images of natural rocks, $S/\psi$ can range from 0.15 (Fountainebleau sandstone) to 0.7 (Kentucky sandstone) when E$(\omega ) = 1$.

### A Constitutive Relation for Reaction at the Darcy Scale

A constitutive relation is an additional equation that specifies properties of a material. For example, the longitudinal dispersivity describes the dispersion of solute introduced by variations in fluid velocities in a porous material. In this section, we develop a constitutive relation that models the average solute concentration of a porous medium with varying reaction rates in pores.

#### First-Order Kinetics

Before we approach the full reactive transport problem—Eq. (37)—we start with a single pore involving only mineral dissolution,

44 $$\begin{aligned} \frac{{\mathrm{d}} C}{{\mathrm{d}} t} = {\text{Da}}(1-C), \quad C(t=0) = C_{0}, \end{aligned}$$

where $C_{0}$ is the initial concentration of the mineral-forming solute in the pore. We consider an aspect ratio, $r/L_{z}$, small enough such that a volume-averaged concentration is representative. Using Eq. (24), the Damköhler number in a cylindrical pore is

45 $$\begin{aligned} {\text{Da}} = \frac{2 \omega }{r} \frac{L_{z}^{2} k_{\mathrm{p}}^{\mathrm{I}}}{D}, \end{aligned}$$

where ${2\omega /r}$ defines the pore-scale geometry and ${L_{z}^{2} k_{\mathrm{p}}^{\mathrm{I}}/D}$ defines the physics of the problem. In this work, we focus on analyzing how pore-scale geometry affects solute concentration and reaction rate over time, assuming constant ${L_{z}^{2} k_{\mathrm{p}}^{\mathrm{I}}/D}$. We abbreviate ${L_{z}^{2} k_{\mathrm{p}}^{\mathrm{I}}/D}$ to ${\text{Da}}_{\mathrm{p}}$. The solution for the single-pore reaction problem is

46 $$\begin{aligned} C(t;\, \omega , r) = (C_{0}-1) e^{(-2 {\text{Da}}_{\mathrm{p}} \omega /r)t} +1. \end{aligned}$$

We define the volume-averaged solution as

47 $$\begin{aligned} C_{\mathrm{avg}}(t)&= \dfrac{\displaystyle \int _{0}^{1} \displaystyle \int _{0}^{\infty } C(t;\, \omega , r) p(\omega , r) r^{2} {\mathrm{d}}r {\mathrm{d}}\omega }{\displaystyle \int _{0}^{\infty } p(r) r^{2} {\mathrm{d}}r } \end{aligned}$$

48 $$\begin{aligned}&= (C_{0} - 1)\dfrac{\displaystyle \int _{0}^{1} \displaystyle \int _{0}^{\infty } e^{(-2 {\text{Da}}_{\mathrm{p}} \omega /r)t} p(\omega , r) r^{2} {\mathrm{d}}r {\mathrm{d}}\omega }{\displaystyle \int _{0}^{\infty } p(r) r^{2} {\mathrm{d}}r } +1. \end{aligned}$$

The bounds of the integral over pore size, *r*, should correspond to the bounds of the prescribed pore-size distribution. Consider $C_{0}=0$ and expand the volume-averaged solution using a Taylor series around $t=0$:

49 $$\begin{aligned} C_{\mathrm{avg}}(t)&= -\sum _{k=0}^{\infty } \left( \frac{1}{k!} \int _{0}^{1} \int _{0}^{\infty } \left( \frac{-2{\text{Da}}_{\mathrm{p}}\omega t}{r} \right) ^{k} p(\omega , r) r^{2} {\mathrm{d}}r {\mathrm{d}}\omega \right) /{{\text{E}}}(r^{2}) +1 \end{aligned}$$

50 $$\begin{aligned}&= -\sum _{k=1}^{\infty } \left( \frac{(-2{\text{Da}}_{\mathrm{p}}t)^{k}}{k!} \int _{0}^{1} \int _{0}^{\infty } p(\omega , r) \omega ^{k} r^{2-k} {\mathrm{d}}r {\mathrm{d}}\omega \right) /{{\text{E}}}(r^{2}) \end{aligned}$$

51 $$\begin{aligned}&= -\sum _{k=1}^{\infty } \left( \frac{(-2{\text{Da}}_{\mathrm{p}}t)^{k}}{k!} \frac{{{\text{E}}}(\omega ^{k} r^{2-k})}{{{\text{E}}}(r^{2})} \right) \end{aligned}$$

52 $$\begin{aligned}&= 2{\text{Da}}_{\mathrm{p}} t\frac{{{\text{E}}}(\omega r)}{{{\text{E}}}(r^{2})} - 2({\text{Da}}_{\mathrm{p}} t)^{2}\frac{{{\text{E}}}(\omega ^{2})}{{{\text{E}}}(r^{2})} + O(t^{3}). \end{aligned}$$

Let us now describe the Darcy-scale reaction in the same manner as we did for a single pore, Eq. (44). In this case, the definition of the Darcy-scale Damköhler number,

53 $$\begin{aligned} {\text{Da}}_{\mathrm{d}} = 2{\text{Da}}_{\mathrm{p}}\frac{{{\text{E}}}(\omega r)}{{{\text{E}}}(r^{2})} \end{aligned}$$

leads to a first-order approximation of the volume-averaged concentration. To better approximate $C_{\mathrm{avg}}$, we propose a nonlinear reaction-rate model as a constitutive relation,

54 $$\begin{aligned} \frac{{\mathrm{d}} {\overline{C}}}{{\mathrm{d}} t} = {\text{Da}}_{\mathrm{d}}(1-{\overline{C}})^n, \quad {\overline{C}}(t=0) = C_{0}, \end{aligned}$$

where *n* is the reaction order (Lasaga 1998). Many researchers have attempted to explain values of the reaction order in terms of dissolution or precipitation processes (Blum and Lasaga 1987; Teng et al. 2000). However, attributions of a process on the basis of this exponent are generally not defensible without further observations (Brantley 2008). For further discussion, see Brantley (2003, 2008).

The solution to the nonlinear reaction-rate model, Eq. (54), is

55 $$\begin{aligned} {\overline{C}}(t) = 1 - \left[ {\text{Da}}_{\mathrm{d}}(n-1)\,t + (1-C_{0})^{1-n} \right] ^{1/(1-n)}. \end{aligned}$$

Its Taylor series expansion around $t=0$ while $C_{0} = 0$ is

56 $$\begin{aligned} {\overline{C}}(t)&= 1 -\sum _{k=0}^{\infty } \frac{\prod _{i=1}^{k-1} \left( i(n-1)+1 \right) }{k!} (-{\text{Da}}_{\mathrm{d}} t)^{k} \end{aligned}$$

57 $$\begin{aligned}&= {\text{Da}}_{\mathrm{d}} t - \frac{n}{2} ({\text{Da}}_{\mathrm{d}} t)^{2} + \frac{n (2n-1)}{6} ({\text{Da}}_{\mathrm{d}} t)^{3} + O(t^{4}). \end{aligned}$$

We observe that the Darcy-scale Damköhler number, Eq. (53), still matches the first-order term of the volume-averaged solution, Eq. (52). If we define

58 $$\begin{aligned} n = \frac{{{\text{E}}}(\omega ^{2}) {{\text{E}}}(r^{2})}{{{\text{E}}}^{2}(\omega r)}, \end{aligned}$$

then the nonlinear reaction-rate model approximates the volume-averaged concentration to the second order with respect to time. By Cauchy–Schwarz inequality, we infer $n \ge 1$, which agrees with experimental observations. The inverse square root of this definition of the reaction order, *n*, is also known as Tucker’s congruence coefficient, which assesses similarity between two variables (Lorenzo-Seva and ten Berge 2006).

**Fig. 1:**
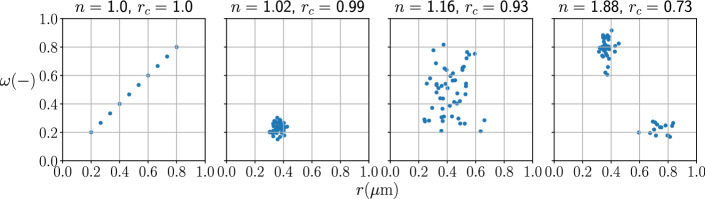
This figure shows scatter plots of pore sizes and effective-surface-area ratio. The title of each plot shows the reaction order, *n*, and Tucker’s congruence coefficient, $r_{\mathrm{c}}$

Figure 1 shows scatter plots of pore sizes and effective-surface-area ratios. Each point represents an observation of the pore size and the effective-surface-area ratio in a porous sample. Tucker’s congruence coefficient, $r_{\mathrm{c}}$, measures the similarity between pore size and effective-surface-area ratio. From the leftmost figure to the rightmost figure, the congruence coefficient decreases as the observations become less similar, or more heterogeneous. Since the reaction order, *n*, is the squared inverse of the congruence coefficient, the reaction order increases as the heterogeneity increases. Such a definition of the reaction order is a function of the geometric variables $\omega$ and *r*. Thus we can use the reaction order to infer pore-scale spatial heterogeneity of minerals.

#### Second-Order Kinetics

We model the single-pore problem with second-order kinetics by

59 $$\begin{aligned} \frac{\mathrm{d} C}{\mathrm{d} t} = {\text{Da}}(1-C^{2}), \quad C(t=0) = 0, \end{aligned}$$

where the solution is

60 $$\begin{aligned} C = \tanh {({\text{Da}} \cdot t)}. \end{aligned}$$

Using Eq. (20), the Damköhler number in a cylindrical pore is

61 $$\begin{aligned} {\text{Da}} = \frac{2 \omega }{r} \frac{L_{z}^{2} \sqrt{k_{\mathrm{d}} \smash [b]{k_{\mathrm{p}}^{\mathrm{II}}}}}{D}, \end{aligned}$$

where ${L_{z}^{2} \sqrt{k_{\mathrm{d}} \smash [b]{k_{\mathrm{p}}^{\mathrm{II}}}}/D}$, which we abbreviate to ${\text{Da}}_{\mathrm{p}}$, defines the physics of the problem for second-order kinetics. The volume-averaged concentration is defined using Eq. (47), and we apply Taylor series expansion to the volume-averaged concentration,

62 $$\begin{aligned} C_{\mathrm{avg}}(t) =\,&\sum _{k=1}^{\infty } \left( \frac{B_{2k} 4^{k} (4^{k} - 1)}{(2k)!} \int _{0}^{1} \int _{0}^{\infty } \left( \frac{2{\text{Da}}_{\mathrm{p}}\omega t}{r} \right) ^{2k-1} p(\omega , r) r^{2} {\mathrm{d}}r {\mathrm{d}}\omega \right) /{{\text{E}}}(r^{2}) \end{aligned}$$

63 $$\begin{aligned} =\,&\sum _{k=1}^{\infty } \left( \frac{B_{2k} 4^{k} (4^{k} - 1) (2 {\text{Da}}_{\mathrm{p}} t)^{2k-1}}{(2k)!} \int _{0}^{1} \int _{0}^{\infty } p(\omega , r) \omega ^{2k-1} r^{3-2k} {\mathrm{d}}r {\mathrm{d}}\omega \right) /{{\text{E}}}(r^{2}) \end{aligned}$$

64 $$\begin{aligned} =\,&2{\text{Da}}_{\mathrm{p}} t \frac{{{\text{E}}}(\omega r)}{{{\text{E}}}(r^{2})} - \frac{(2{\text{Da}}_{\mathrm{p}} t)^{3}}{3} \frac{{{\text{E}}}(\omega ^{3} r^{-1})}{{{\text{E}}}(r^{2})} + O(({\text{Da}}_{\mathrm{p}} t)^{5}), \end{aligned}$$

where $B_{2k}$ is the Bernoulli number (Oldham et al. 2009). The Taylor series expansion of the hyperbolic tangent function, Eq. (60), converges for ${{\text{Da}}\, t < \pi /2}$, which is not of concern since we utilize only the derivatives of ${C_{\mathrm{avg}}(t=0)}$.

We propose the following constitutive relation that describes the solute concentration at the Darcy scale,

65 $$\begin{aligned} \frac{{\mathrm{d}} {\overline{C}}}{{\mathrm{d}} t} = {\text{Da}}_{\mathrm{d}}(1-{\overline{C}}^{2})^n, \quad {\overline{C}}(t=0) = 0. \end{aligned}$$

We do not attempt a solution for ${\overline{C}}$. However, we can still expand ${\overline{C}}$ around $t=0$ with Taylor series:

66 $$\begin{aligned} {\overline{C}} = {\text{Da}}_{\mathrm{d}} t - \frac{n(n+1)}{6} ({\text{Da}}_{\mathrm{d}} t)^{3} + O(t^{3} ). \end{aligned}$$

See “Appendix 2” for detailed derivations. Comparing the third-order term of Eq. (64) with that of Eq. (66) yields

67 $$\begin{aligned} \frac{n(n+1)}{2} = \frac{{\text{E}}(\omega ^{3} r^{-1}) {\text{E}}^{2}(r^{2})}{{\text{E}}^{3}(\omega r)}. \end{aligned}$$

One can utilize the quadratic formula to explicitly determine *n*,

68 $$\begin{aligned} n = \left( -1+\sqrt{1+8{\text{E}}(\omega ^{3} r^{-1}) {\text{E}}^{2}(r^{2})/{\text{E}}^{3}(\omega r) } \right) /2, \end{aligned}$$

where we consider only the larger value of *n* as a solution. Note that the reaction orders for first- and second-order kinetics are non-dimensional and, most importantly, independent of the length scale and the reaction rate constants.

### Goodness of Fit Between the Pore-Scale and the Darcy-Scale Concentrations

There exists a variety of goodness-of-fit measures between models and experimental observations. For example, the coefficient of determination, $R^{2}$, is often used to determine the kinetic rate law when applying the integral method (Brantley and Conrad 2008; Zhao and Skelton 2014). The mean-squared error is also a goodness-of-fit measure, and the least-squares approach tends to minimize such a metric. We use another goodness-of-fit measure, the Jensen–Shannon divergence, which is based on the Kullback–Leibler divergence (Kullback and Leibler 1951). The Kullback–Leibler divergence between some unknown distribution, *p*(*x*), and an approximating distribution, *q*(*x*), is:

69 $$\begin{aligned} {\text{KL}}(p\Vert q) = \int p(x) \log _{2}{\left( \frac{p(x)}{q(x)} \right) } {\mathrm{d}}x. \end{aligned}$$

The Kullback–Leibler divergence satisfies ${\text{KL}}(p\Vert q) \ge 0$ with equality if, and only if, $p(x)=q(x)$ (Bishop 2006). Although ${\text{KL}}(p\Vert q) \ge 0$, it may diverge to infinity depending on the underlying densities (Nielsen 2020). Thus we use the Jensen–Shannon divergence,

70 $$\begin{aligned} {\text{JS}}(p\Vert q) =\,&\frac{1}{2} \left( {\text{KL}}\left( p\Vert \frac{p+q}{2}\right) + {\text{KL}}\left( q\Vert \frac{p+q}{2}\right) \right) \end{aligned}$$

71 $$\begin{aligned} =\,&\frac{1}{2} \int \left( p(x) \log _{2}{\left( \frac{2 p(x)}{p(x) + q(x)} \right) } + q(x) \log _{2}{\left( \frac{2 q(x)}{p(x) + q(x)} \right) } \right) {\mathrm{d}}x, \end{aligned}$$

which is bounded between 0 and 1 when using base-2 logarithms (Lin 1991). Throughout this work, we use the Jensen–Shannon distance, which is defined as the square root of the Jensen–Shannon divergence.

Such a metric measures the distance between probability distributions (Endres and Schindelin 2003; Österreicher and Vajda 2003; Levene and Kononovicius 2019). The following describes how we apply this measure to solute concentration over time, $C_{\mathrm{avg}}(t)$ and ${\overline{C}}(t)$. Suppose we regard solute concentration over time as cumulative distribution functions. In that case, we measure the Jensen–Shannon divergence of their derivatives, which can be seen as the probability density functions or the reaction rates over time.

When the observed solute concentration is not monotonically increasing over time like a cumulative distribution function, we simply use the root mean square error (RMSE) as a quality measure of the constitutive relation,

72 $$\begin{aligned} {\mathrm{RMSE}} = \sqrt{\dfrac{\displaystyle \int (p(x) - q(x))^{2} {\mathrm{d}}x}{\max {(x)}-\min {(x)}}}. \end{aligned}$$

### Determination of the Darcy-Scale Damköhler Number, ${\text{Da}}_{\mathrm{d}}$, and the Reaction Order, *n*, using power series

We test the constitutive relation using the volume-averaged concentration $C_{\mathrm{avg}}(t)$, which is an analogy of solute concentration measurement from a dissolution experiment. Assume the solute concentration can be described by the constitutive relation within a certain error. Then we can use the Taylor series expansions, Eqs. (57) and (66), to obtain the Darcy-scale Damköhler number,

73 $$\begin{aligned} {\text{Da}}_{\mathrm{d}} = C_{\mathrm{avg}}^{'}(t=0), \quad C_{\mathrm{avg}}(t=0)=0, \end{aligned}$$

which is the initial rate of reaction. When the kinetics is of first order, the reaction order is obtained by differentiating Eq. (57) twice,

74 $$\begin{aligned} n = -\frac{C_{\mathrm{avg}}^{''}(t=0)}{({\text{Da}}_{\mathrm{d}})^{2}}. \end{aligned}$$

Differentiating Eq. (66) thrice and rearranging yields the reaction order for second-order kinetics,

75 $$\begin{aligned} n(n+1) = -\frac{C_{\mathrm{avg}}^{'''}(t=0)}{({\text{Da}}_{\mathrm{d}})^{3}}. \end{aligned}$$

This method of determining ${\text{Da}}_{\mathrm{d}}$ and *n* utilizes power-series expansion and requires only the derivatives of concentration at $t=0$, given $C_{\mathrm{avg}}(t=0)=0$.

**Fig. 2:**
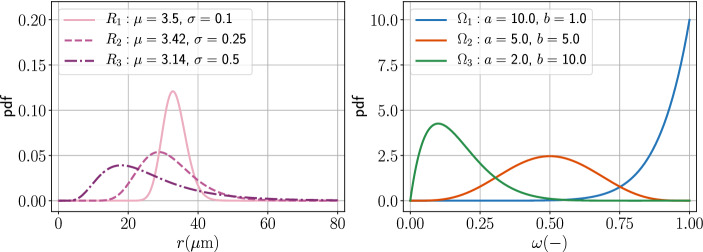
This figure shows the probability density functions of the distribution of pore sizes and effective-surface-area ratio. The legends state the essential parameters for generating the probability density functions. The definitions of the probability density functions are defined in Eqs. (38) and (42)

We consider three sets of log-normally distributed pore sizes, $R_{1}$, $R_{2}$, and $R_{3}$, which have $S/\psi \approx 0.6$ but different variances. The pore sizes are chosen such that they range from 10 to 80 $\upmu$m (Gong et al. 2020). Likewise, we assume the effective surface area follows the beta distribution, where $\Omega _{1}$ considers most pores are fully reactive, $\Omega _{2}$ assumes a larger variance of mineral surface area in the pores, and $\Omega _{3}$ implies that the reactive mineral constitutes a small portion of the porous sample. Figure 2 shows the details of the aforementioned probability distributions.

The products of the random variables *R* and $\Omega$ form nine scenarios of the bundle-of-tubes model, which can be used as benchmarks for our power-series approach to obtain the Darcy-scale Damköhler number and the reaction order. We compare this method with a goodness-of-fit minimization using both ${\text{Da}}_{d}$ and *n* as unknowns, similar to the ideas of nonlinear least-squares model fitting (Fogler 2016). Initially, the pores are filled with dissolving fluid with no solute concentration, ${C=0}$. Then the mineral starts to dissolve into the fluid, such that the solute concentration increases. We assume we can observe the average concentration, $C_{\mathrm{avg}}$, without transport effects. To capture the full reaction behavior, the simulation ends when concentration $C_{\mathrm{avg}}$ is larger than 0.99. The physics-related parameters, ${\text{Da}}_{\mathrm{p}}$, is set as 50, such that the Darcy-scale Damköhler numbers of the scenarios are at a similar scale.

### Flow-Through Experiment

In the previous section, we test the constitutive relation considering only mineral reaction. To measure the solute concentration of the fluid in a porous sample, one has to push the fluid out of the porous sample. We now discuss the full reactive transport problem. Consider a flow-through experiment, i.e., injecting fluid that dissolves the mineral in a porous sample. We collect the fluid from the outlet and measure the solute concentration over time. Before the experiment, the porous sample should be saturated by the dissolving fluid, which will result in an initial concentration of the solute in the fluid. Then we start the flow-through experiment by injecting fluid without solute under high Péclet number. This process aims to flush out all residual concentrations, such that the initial condition of the concentration corresponds to the upscaling approach considering only reaction. We take fluid samples from the outlet and measure the solute concentration. Since we are injecting fluid with zero solute concentration, we expect the measured solute concentration at the outlet to decrease over time. When the outlet concentration is close to zero, we reduce the Péclet number to 0.1–1% of the original Péclet number in order to observe an increase of the outlet solute concentration. The flow-through experiment creates a V-shaped curve of outlet concentration over time.

We use FEniCS (Alnaes et al. 2015) to solve the transient initial boundary value problem of each pore and apply Eq. (47) to obtain the average concentration. We discuss two types of porous samples, namely the combinations of the pore-size distribution and the effective-surface-area ratio, $R_{1} \Omega _{3}$ and $R_{3} \Omega _{3}$. We test the porous samples for the low and high Damköhler-number cases, described in the following subsections. In all cases, we consider the molecular diffusivity, *D*, as $5\times 10^{-9}$ m$^{2}$ s$^{-1}$.

#### The Low Damköhler-Number Case, ${\text{Da}}_{\mathrm{d}} = 0.1$

The Darcy-scale Damköhler number, Eq. (53), is proportional to the length scale squared. This case is suitable when the mineral has low reaction rates or when the porous domain is short (small length scale), e.g., a 5  cm rock sample in a laboratory. We consider this as our “small length-scale scenario” for which the outlet solute concentration can be measured. Since the Damköhler number is low, the solute concentration during injection of the dissolving fluid should be far from chemical equilibrium. Therefore, we assume the reaction is of first order.

Following the procedure of the flow-through experiment, we set the initial Péclet number as 10, and the Darcy-scale Damköhler number is 0.1. We reduce the Péclet number to 0.01 at 0.15 nondimensional time. The simulation ends at 1.5 nondimensional time, which for the 5 cm rock sample mentioned above would correspond to about 8.7 days.

The solute concentration over time at the outlet of the porous sample is collected from the simulation data. Knowing the Péclet number and that the mineral reaction is of first order, we fit the observed concentration over time with the reactive transport model,

76 $$\begin{aligned} \frac{\partial {\overline{C}}}{\partial t} + {\text{Pe}}_{\mathrm{d}} \frac{\partial {\overline{C}} }{\partial z} - \left( 1 + ({\text{Pe}}_{\mathrm{d}})^{2} t\, f(r) \right) \frac{\partial ^{2} {\overline{C}}}{\partial z^{2}} = {\text{Da}}_{\mathrm{d}}(1-{\overline{C}})^n. \end{aligned}$$

The shape factor, *f*(*r*), is defined using Eq. (35). Utilizing the optimization procedures in SciPy (Virtanen et al. 2020), we find ${\text{Da}}_{\mathrm{d}}$ and *n* by minimizing the RMSE between the observation and the model.

Another method of fitting ${\text{Da}}_{\mathrm{d}}$ and *n* is to utilize a part of ${C_{\mathrm{avg}}(t)}$, where the diffusion effects are dominant enough (${{\text{Pe}}_{\mathrm{d}} \ll 1}$) such that we can treat the concentration as constant over space. Owing to the divergence theorem and the boundary condition ${C(0,t)=0}$ results in

77 $$\begin{aligned} \frac{\partial {\overline{C}} }{\partial t} + {\text{Pe}}_{\mathrm{d}} {\overline{C}}(1,t) = {\text{Da}}_{\mathrm{d}} (1-{\overline{C}})^n. \end{aligned}$$

**Fig. 3:**
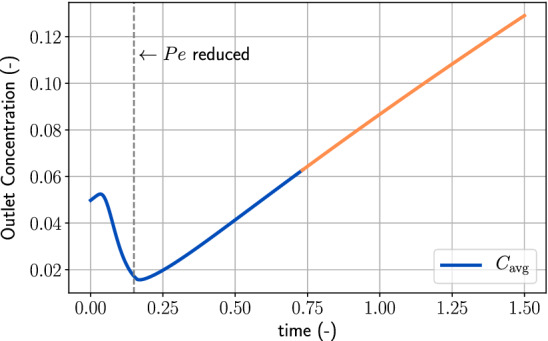
The outlet concentration over time of the low Damköhler-number problem. The orange line shows the part where we apply the least-squares fitting technique Eq. (77). The vertical dashed line indicates the time, $t=0.15$, when the injection rate is reduced

Then, we can perform the least-squares fitting on the left-hand side to determine ${\text{Da}}_{\mathrm{d}}$ and *n*. Figure 3 shows the outlet concentration of the flow-through experiment and highlights the region in which we consider diffusion to be dominant. Such an approach relies heavily on the strong-diffusion assumption and is therefore not suitable for the high Damköhler-number case, discussed in the next section.

#### The High Damköhler-Number Case, ${\text{Da}}_{\mathrm{d}}={4\times 10^{4}}$

Consider a field experiment in an aquifer, where the length scale is on the order of meters. Here, a fluid is injected in one well and produced at another well. In this case, it may not be possible to observe a concentration breakthrough at the outlet. Thus, we perform simulations of a flow-through experiment and measure the concentration at the inlet over time. The Darcy-scale Damköhler number is $4\times 10^{4}$. We assume a second-order kinetics model for the mineral reaction, and our reactive transport model is

78 $$\begin{aligned} \frac{\partial {\overline{C}}}{\partial t} + {\text{Pe}}_{\mathrm{d}} \frac{\partial {\overline{C}} }{\partial z} - \left( 1 + ({\text{Pe}}_{\mathrm{d}})^{2} t\, f(r)\right) \frac{\partial ^{2} {\overline{C}}}{\partial z^{2}} = {\text{Da}}_{\mathrm{d}}(1- {\overline{C}}^{2})^n. \end{aligned}$$

We consider three cases of initial Péclet numbers, $4\times 10^{3}$, ${8\times 10^{3}}$, and ${8\times 10^{4}}$. The injection stops at ${2\times 10^{-6}}$ dimensionless time, and the simulation ends at ${1\times 10^{-5}}$ dimensionless time, which is roughly 231.5 days considering a 100 m simulation domain.

Though it is practically not possible to observe the inlet concentration during the injection phase, we perform the fitting of ${\text{Da}}_{\mathrm{d}}$, *n*, and *f*(*r*) using all information of $C_{\mathrm{avg}}$ at the inlet. In the latter phase when injection stops (rising limb of the curve in Fig. 3), the concentration at the inlet can be determined by sampling the fluid in the injection well.

## Results

In this section, we show the benchmarks of the power-series approach and the simulations of flow-through experiments. Then, we discuss the results in Sect. 4.

### Benchmark of the Power-Series Approach

We benchmark the power-series approach that obtains the reaction order, *n*, and the Darcy-scale Damköhler number, ${\text{Da}}_{\mathrm{d}}$, using a bundle of tubes characterized by the distributions of pore sizes and effective-surface-area ratio described in Sect. 2.6.

**Fig. 4:**
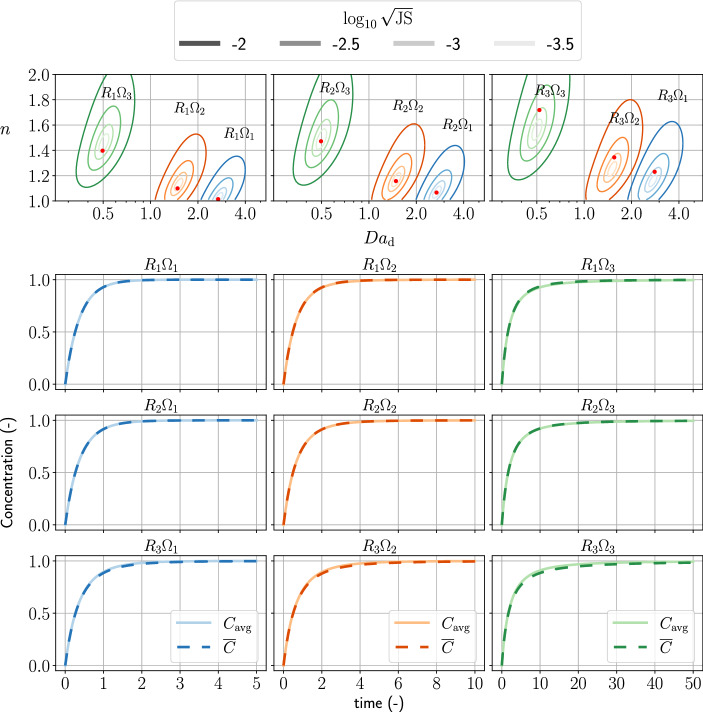
The upper part of the figure shows the contours of the log-scaled Jensen–Shannon distance between the observed concentration and the modeled concentration of first-order reactions using the constitutive relation. The red points are the Darcy-scale Damköhler number and the reaction order approximated by the power-series approach (Eqs. (73) and (74)). The legend shows the value of the log-scaled Jensen–Shannon distance using a grayscale, corresponding to the brightness of the colored contour lines. The lower part of the figure shows the concentrations over time $C_{\mathrm{avg}}$ and ${\overline{C}}$ of all pore sizes and effective-surface-area ratios scenarios

**Fig. 5:**
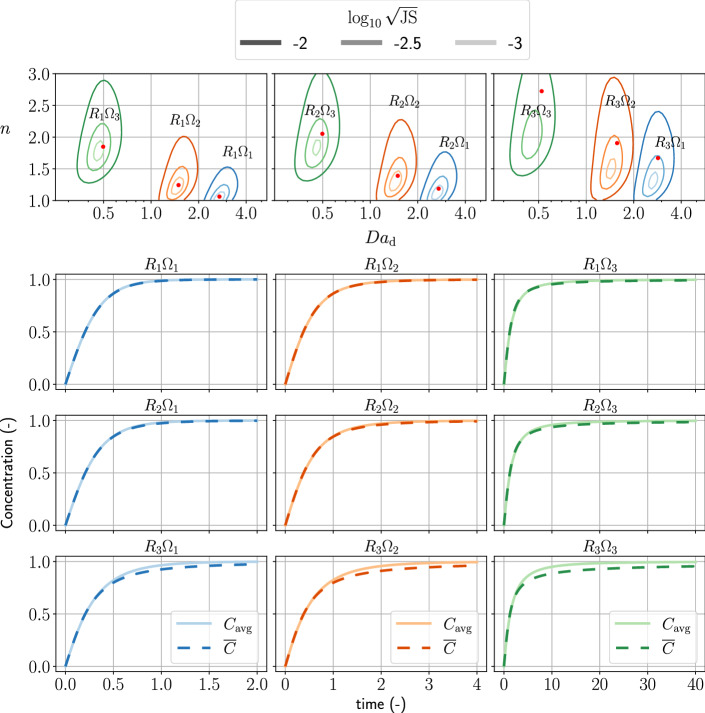
The upper part of the figure shows the contours of the log-scaled Jensen–Shannon distance between the observed concentration and the modeled concentration of second-order kinetics using the constitutive relation. The red points are the Darcy-scale Damköhler number and the reaction order approximated by the power series approach [Eqs. (73) and (75)]. The legend shows the value of the log-scaled Jensen–Shannon distance using a grayscale, corresponding to the brightness of the colored contour lines. The lower part of the figure shows the concentrations over time $C_{\mathrm{avg}}$ and ${\overline{C}}$ of all pore sizes and effective-surface-area ratios scenarios

Figures 4 and 5 show the benchmark of the first-order kinetics and second-order kinetics, respectively. In the upper part of the figures, we plot the contour lines of log-scaled Jensen–Shannon distance between $C_{\mathrm{avg}}$ and ${\overline{C}}$. The red points indicate the approximation of ${\text{Da}}_{\mathrm{d}}$ and *n* using the power-series approach. In an ideal case, the red points should be in the minimum of the Jensen–Shannon distance. In the lower part of the figures, we plot the concentration over time of all scenarios.

**Fig. 6:**
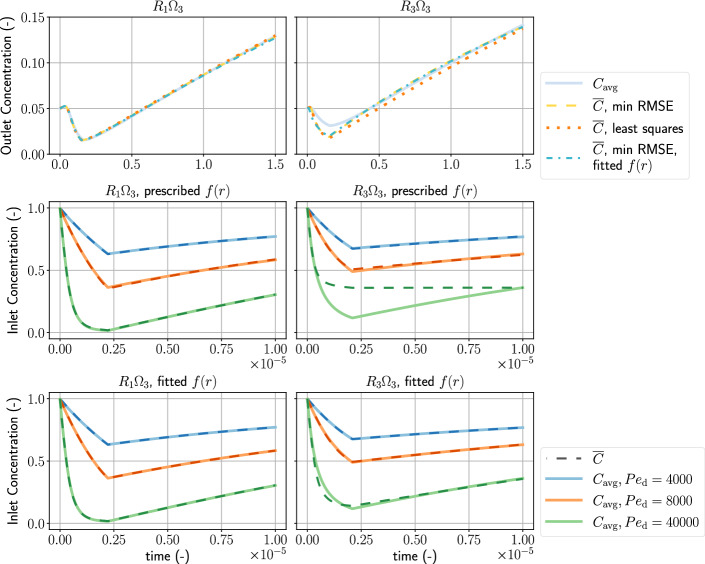
The figures show the concentration $C_{\mathrm{avg}}$ (solid lines) and the modeled concentration ${\overline{C}}$ (dashed lines) of the low Damköhler-number cases on the top panel. The high Damköhler-number cases are summarized in the middle and the bottom panel. The left and right panels show the $R_{1} \Omega _{3}$ and the $R_{3} \Omega _{3}$ cases, respectively

### Flow-Through Experiment

Figure 6 shows the observed concentration, $C_{\mathrm{avg}}$, and the modeled concentration, ${\overline{C}}$. Table 1 shows the Darcy-scale Damköhler number, the reaction order, and shape factor obtained by direct calculation [Eqs. (53), (58), and (68)], and by RMSE minimization of the low and high Damköhler-number cases (${\text{Da}}_{\mathrm{d}}=0.1$ and ${\text{Da}}_{\mathrm{d}}=40{,}000$, respectively) corresponding to the $R_{1}\Omega _{3}$ and the $R_{3} \Omega _{3}$ distributions. In the low Damköhler-number scenarios, we use the least-squares curve fitting method, Eq. (77), to obtain ${\text{Da}}_{\mathrm{d}}$ and *n*. The results of the least-squares curve fitting are ${\text{Da}}_{\mathrm{d}}=0.1$, $n=1.38$ for the $R_{1} \Omega _{3}$ scenario, and ${\text{Da}}_{\mathrm{d}}=0.1$, $n=1.63$ for the $R_{3} \Omega _{3}$ scenario.

**Table 1:** This table summarizes the Darcy-scale Damköhler number, ${\text{Da}}_{\mathrm{d}}$, the reaction order, *n*, and the shape factor, *f*(*r*), obtained by fitting the concentration-over-time curve using the constitutive relation with the RMSE metric

*R*_3_Ω_3_				
Initial ${\text{Pe}}_{\mathrm{d}}$	10	4000	8000	40,000
Direct calculation				
${\text{Da}}_{\mathrm{d}}$	0.1		40,000	
*n*	1.40		1.77	
*f*(*r*)		$blank$	$4.081\times 10^{-2}$	
Minimum RMSE				
${\text{Da}}_{\mathrm{d}}$	0.1	52,528	45,154	38,464
*n*	1.57	1.57	1.66	1.30
Minimum RMSE with shape factor fitting				
${\text{Da}}_{\mathrm{d}}$	0.1	50,887	42,049	38,950
*n*	1.68	1.55	1.59	1.53
*f*(*r*)	$2.39\times 10^{-2}$	$1.402\times 10^-1$	$1.222\times 10^{-1}$	$3.168\times 10^{-2}$
$$R_{3} \Omega _{3}$$				
Direct calculation				
${\text{Da}}_{\mathrm{d}}$	0.1		40,000	
*n*	1.74		2.60	
*f*(*r*)			1.718	
Minimum RMSE				
${\text{Da}}_{\mathrm{d}}$	0.13	25,376	24,691	$10^{-6}$*
*n*	4.60	1.0	1.43	4.89*
Minimum RMSE with shape-factor fitting				
${\text{Da}}_{\mathrm{d}}$	0.12	35,791	25,025	29,111
*n*	3.87	1.56	1.0	1.0
*f*(*r*)	16.8	2.091	1.433	0.463

The values of the direct calculation are the result of prescribing ${\text{Da}}_{\mathrm{p}}$, the $R_{1} \Omega _{3}$ distribution, and the $R_{3} \Omega _{3}$ distribution. In the low ${\text{Da}}_{\mathrm{d}}$ case, we assumed first-order kinetics. Hence we use Eq. (58) to calculate the reaction order. We assumed second-order kinetics for the high ${\text{Da}}_{\mathrm{d}}$ case, and Eq. (68) is used for calculating the reaction order for second-order kinetics. The direct calculation of *f*(*r*) uses Eq. (41). The results of the least-squares curve fitting are ${\text{Da}}_{\mathrm{d}}=0.1$, $n=1.38$ for the $R_{1} \Omega _{3}$ scenario, and ${\text{Da}}_{\mathrm{d}}=0.1$, $n=1.63$ for the $R_{3} \Omega _{3}$ scenario

*The fitting in this case does not yield reasonable results

## Discussion

### Benchmark of the Power-Series Approach

In Fig. 4, we observed that the power-series approach obtains ${\text{Da}}_{\mathrm{d}}$ and *n* close to the minimum Jensen–Shannon distance. The connections between nonlinear fitting of the parameters, ${\text{Da}}_{\mathrm{d}}$ and *n*, and the geometric information of the porous medium are established since the power-series approach is exact for retrieving expected values of the pore-size and the effective-surface-area distributions.

In Fig. 5, we observed general agreement of ${\text{Da}}_{\mathrm{d}}$ and *n* obtained by the power-series approach to those at the minimum Jensen–Shannon distance. As the variance of the pore-size increases, the obtained ${\text{Da}}_{\mathrm{d}}$ and *n* (red points) stray from the minimum Jensen–Shannon distance. In the concentration plots of the $R_{3} \Omega _{1}$, $R_{3} \Omega _{2}$, and $R_{3} \Omega _{3}$ cases, the modeled concentration fits well when ${{\overline{C}}<0.5}$. Some discrepancy between ${\overline{C}}$ and $C_{\mathrm{avg}}$ is present when ${{\overline{C}}>0.5}$. Comparing to the results of first-order kinetics, where ${\overline{C}}$ fits $C_{\mathrm{avg}}$ well throughout all concentrations, our averaged model of second-order kinetics can only fit reactions far from equilibrium (e.g., ${{\overline{C}}<0.5}$).

The power-series approach of obtaining ${\text{Da}}_{\mathrm{d}}$ and *n* suffers from the fact that:

1. reaction rates at zero concentration can be hard to obtain, and
2. numerical differentiation of higher-order derivatives can yield spurious results.

Therefore, we require other nonlinear fitting methods by minimizing the divergence between models and observations. In the next section, we discuss the intricacies of nonlinear fitting of solute concentration during flow-through experiments.

### Flow-Through Experiment

#### The $R_{1} \Omega _{3}$ Scenario

The top-left panel of Fig. 6 shows the low Damköhler-number, first-order-kinetics case, where both the RMSE minimization and the least-squares fitting method fit the outlet concentration $C_{\mathrm{avg}}$. Both methods of obtaining ${\text{Da}}_{\mathrm{d}}$ and *n* are accurate within 15% relative error as confirmed by direct calculation from pore-size and effective-surface-area distributions. The good agreement can be attributed to the fact that the solute can be mostly flushed out from the porous domain, due to the low Damköhler number. Hence, the solute concentration in each tube goes down to almost zero, and spatial gradients of the solute concentration in the *z* direction are negligible. This creates a situation similar to the problem considering only reaction effects, which is not the case for the scenarios of high Damköhler-number, second-order kinetics.

Focusing on the high Damköhler-number scenarios, we observe general agreement of fitted parameters. For the case of initial ${\text{Pe}}_{\mathrm{d}}=4000$, the error in ${\text{Da}}_{\mathrm{d}}$ can be attributed to the fact that the volume injected is not enough, such that the dissolution in smaller pores is not observed in $C_{\mathrm{avg}}$. The case of initial ${\text{Pe}}_{\mathrm{d}}=8000$ approximated ${\text{Da}}_{\mathrm{d}}$ closer to the prescribed value than the previous case. The third case of initial ${\text{Pe}}_{\mathrm{d}}=40{,}000$, performed the best in retrieving ${\text{Da}}_{\mathrm{d}}$. This may be caused by, similar to the low Damköhler-number case, that the initial solute concentration of all pores is pushed out from the porous domain, such that $C_{\mathrm{avg}}$ is close to zero. The purpose of comparing these three cases is to emphasize that the inversion of parameters is influenced by how we perform the injection test, namely, by the selection of the initial Péclet number.

Furthermore, we performed a fitting in all cases considering the shape factor as unknown. For the low Damköhler number case, we have a good fit of *f*(*r*) within an order of magnitude. However, the obtained value for *n* exhibits a bigger error. This signals the vagueness of the reaction order and the shape factor in minimizing RMSE, where increasing or decreasing either one of the variables leads to similar RMSE. For the high Damköhler number cases, the obtained ${\text{Da}}_{\mathrm{d}}$ improved slightly comparing to the cases with a prescribed shape factor. All fitted shape factors are within an order of magnitude compared to the theoretical calculations. In particular, for the case of initial ${\text{Pe}}_{\mathrm{d}}=40{,}000$, we observe a good fit of the retrieved shape factor.

#### The $R_{3} \Omega _{3}$ Scenario

This scenario tests how effective the pore structure can be inferred in a more dispersive setup. Table 1 summarizes the obtained ${\text{Da}}_{\mathrm{d}}$ and *n* for both the low and high Damköhler-number cases. For the low Damköhler-number case, the minimum RMSE methods do not yield decent approximations of ${\text{Da}}_{\mathrm{d}}$, *n*, and *f*(*r*). The top right panel of Fig. 6 shows that all methods failed to fit the V-shaped region ($t < 0.3$), where dispersive effects matter the most. The pore-size distribution $R_{3}$ mainly consists of smaller pores, as compared to $R_{1}$, which means that the solute to be harder to flush out. This dispersion effect is also demonstrated in the right panel of Fig. 8, where the dispersion model fails to capture the long-tailed concentration profile. However, the least-squares fitting method yields exact ${\text{Da}}_{\mathrm{d}}$, and reasonable *n* within 10% relative error. The least-squares fitting method performs well for the low Damköhler number in both the $R_{1} \Omega _{3}$ and $R_{3} \Omega _{3}$ cases.

For the high Damköhler-number scenarios, fitting the averaged concentration using the model, Eq. (78), does not recover pore-scale information in general. In Fig. 6, the concentration profile of the initial ${\text{Pe}}_{\mathrm{d}}=40{,}000$ with a prescribed shape factor does not fit the averaged concentration. Compared to the results with shape-factor fitting, the prescribed shape factor yields too much dispersion for the given Péclet number. Recall that the longitudinal dispersion coefficient is defined as $({\text{Pe}}_{\mathrm{d}})^{2}t\,f(r)$.

**Fig. 7:**
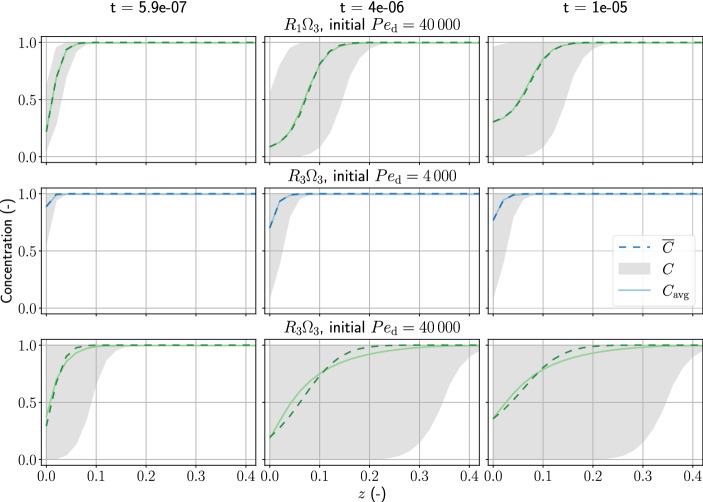
This figure shows the concentration of the pores, *C*, the averaged concentration, $C_{\mathrm{avg}}$, and the modeled concentration, ${\overline{C}}$, of the selected scenarios with shape factor fitting. The left panel shows the solute concentration in the porous domain during fluid injection. The middle panel shows the solute concentration after fluid injection has stopped. The right panel shows the last time step of the simulation, as indicated by the increasing inlet concentration

**Fig. 8:**
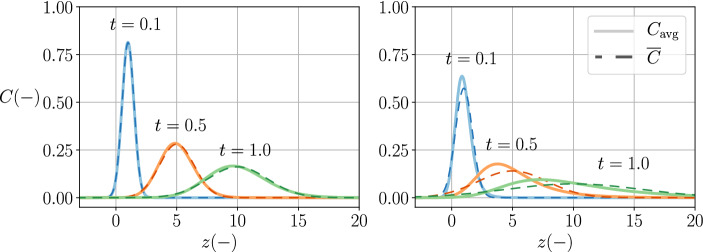
Comparison of the volume-averaged solute concentration and the modeled concentration. The left panel shows the case of pore size distribution $R_{1}$, and the right panel shows the case of pore size distribution $R_{2}$

The case of initial ${\text{Pe}}_{\mathrm{d}}=4000$ with shape-factor fitting gives us the best result in retrieving the model parameters. As shown in both Figs. 6 and 7, the initial Péclet number is not enough to flush out the solute in all pores. However, the dispersion effect is not as pronounced as the initial ${\text{Pe}}_{\mathrm{d}}=40{,}000$ case, due to the lower initial Péclet number. The key to better knowledge of the model parameters and pore-scale information is to control the initial Péclet number for less dispersion. We demonstrate that the rate of injection influences the parameter fitting of the averaged model, due to dispersion caused by the variance of pore sizes. How to properly choose the initial injection rate for inference of pore-scale information could be the subject of future studies.

Figure 7 shows the concentration in pores, the averaged concentration, and the modeled concentration of the high Damköhler number scenarios. When the injection stops, the increase of the inlet solute concentration is not only due to the reaction, but also due to the diffusion of solute from the reservoir to the inlet. We attribute the underestimated *n* to our reactive transport model, Eq. (78), not being able to capture the averaged diffusion effects in each pore, which results in a lower reaction order. Certainly, this situation is not as ideal as the low Damköhler-number situation, which decouples transport and reaction by flushing out almost all of the residual concentration. The modeled concentration, ${\overline{C}}$, still represents the average behavior of the pore concentrations.

To summarize, although imperfect, our method of parameter estimation using a solute concentration breakthrough curve is useful for modeling the average behavior of reactive transport in porous media. The results suggest it is possible to infer pore-scale information using the inversion of averaged parameters.

In all of the flow-through experiments, we considered only one observation point, either the fluid inlet or the fluid outlet. This work serves as a demonstration of the base case with only one observation. To improve the fitting of the reaction order of the high Damköhler-number cases (especially the ones with $R_{3} \Omega _{3}$ distribution), one can incorporate more observation points, spatially distributed within the domain.

### The Applicability of the Constitutive Relations

By adding an exponent to the pore-scale reaction model, the Darcy-scale reaction model effectively describes the averaged behavior of reactions taking place independently in different pores of the porous medium under a specific flow-through experiment. We examine the applicability of the simple approach using the averaged concentration of first-order kinetics as an example. The averaged concentration, Eq. (48), can be considered as a continuous mixture of exponential distributions,

79 $$\begin{aligned} C_{\mathrm{avg}}(t) = 1 - \int _{0}^{\infty } e^{-\lambda t}p(\lambda ) {\mathrm{d}}\lambda , \end{aligned}$$

where $\lambda$ is a parameter that characterizes the exponential distributions. The finite mixture is known as the hyperexponential distribution, which is utilized for fitting long-tail distributions (Feldmann and Whitt 1998; Okada et al. 2020). If $\lambda$ is a gamma distribution, then $C_{\mathrm{avg}}(t)$ is a Pareto distribution (Balakrishna and Lai 2009). The concentration of our proposed model, Eq. (55), has the following form when $C_{0}=0$,

80 $$\begin{aligned} {\overline{C}}(t) = 1 - \left[ 1 + {\text{Da}}_{\mathrm{d}}(n-1)\,t\, \right] ^{1/(1-n)}, \end{aligned}$$

which is the cumulative distribution function of the Pareto distribution, also called the Lomax distribution. There exists a particular ratio distribution of the effective surface area and the pore sizes, ${\Omega /R}$, that satisfies ${\overline{C}}=C_{\mathrm{avg}}$. Such an existence contributes to the effectiveness of the constitutive relation for the first-order kinetics.

In contrast, the theoretical basis of constitutive relations for second-order kinetics is less distinct. We simply followed the derivations of first-order kinetics and exploited the oddity of $C_{\mathrm{avg}}$ and ${\overline{C}}$ to obtain a second-order approximation. The solution for second-order kinetics in a single pore, Eq. (60), can be recast into

81 $$\begin{aligned} C = \tanh {({\text{Da}}\cdot t)} = \dfrac{1-e^{-2{\text{Da}} \cdot t}}{1+e^{-2{\text{Da}} \cdot t}} , \end{aligned}$$

which is a cumulative distribution function of a scaled logistic distribution. If we consider time a semi-infinite domain $[0, \infty )$, such a distribution is also known as the half logistic distribution (Balakrishnan 1985). Though we did not find or derive the relationships of the logistic distribution mixture, we denote the possibility of approximating such a mixture using the Pareto distribution, Eq. (80), since the hyperbolic tangent function can be represented by a Laurent series

82 $$\begin{aligned} \tanh {(t)} = 1 - 2 \sum _{k=0}^{\infty } (-1)^{k} e^{-2t(k+1)}, \; t>0, \end{aligned}$$

which is a mixture of exponential distributions. Such expansion techniques would avoid relying on derivatives around $C=0$ as is done in this study, which may be advantageous but requires more research.

### Limitations and Outlook

The main limitation of this study is that the reaction model we considered is simple and may not be adequate to describe complex geochemical processes, e.g., a rock sample which consists of multiple dissolving minerals. We also assumed the amount of dissolved mineral is minimal such that the geometry of the pores is not affected. Though we considered two common models of first- and second-order kinetics, we treat the rate constant, $k_{\mathrm{d}}$ and $k_{\mathrm{p}}$, as constant, and indeed, $k_{\mathrm{d}}$ and $k_{\mathrm{p}}$ may change as temperature, pH, or ionic strength changes.

Moreover, the assumption that chemical reactions in each tube occur independently of the other tubes is idealized. A better approach is utilizing pore-network models. In pore-network modeling, the porous medium is discretized as a network of pore bodies and pore throats, and the coordination number is defined as number of connections to each pore body. Experimental studies have reported that the average coordination number of a sandstone is $\sim 4$ (Ioannidis and Chatzis 2000; Øren and Bakke 2003; Hefny et al. 2020). Our bundle-of-tubes model has an average coordination number of 0 (or 1, if one considers the pore bodies reside at the fluid inlet and outlet boundaries), which is the base case for pore-network modeling. For this base case, the reaction order does not depend on ${\text{Pe}}_{\mathrm{d}}$ or ${\text{Da}}_{\mathrm{d}}$. The dependence of the reaction order on ${\text{Pe}}_{\mathrm{d}}$ or ${\text{Da}}_{\mathrm{d}}$ for larger coordination numbers requires further studies.

Nonetheless, our simple model reveals a possible mechanistic explanation to the usage of the Darcy-scale reaction order larger than one, and how it can reveal geometric information of the porous medium using the solute breakthrough curve. We suggest considerations of the aforementioned limitations as potential topics for future research.

We propose another possible application of this work in view of energy conservation in a porous sample, where there is only one definition of temperature of the fluid, $T_{\mathrm{f}}^{*}$ and the porous solid, $T_{\mathrm{s}}^{*}$. A special case of energy conservation without pressure work and viscous heating is analogous to Eq. (13):

83 $$\begin{aligned} \frac{\partial T_{\mathrm{f}}^{*}}{\partial t^{*}} + \langle u_{z} \rangle \frac{\partial T_{\mathrm{f}}^{*}}{\partial z^{*}} - D_{\mathrm{f}}\frac{\partial ^{2} T_{\mathrm{f}}^{*}}{\partial z^{*2}} = q_{T}, \end{aligned}$$

where $D_{\mathrm{f}}$ is the thermal diffusivity of the fluid, and $q_{T}$ is a heat source introduced by the porous solid. Usually, such an energy conservation model of heat tracer tests assumes thermal equilibrium between the fluid and the porous solid, $T_{\mathrm{f}}=T_{\mathrm{s}}$, (Shook 2001; Anderson 2005; Saar 2011). However, studies and modeling on thermal disequilibrium between fluid and solid phases have gained interests lately (Karani and Huber 2017; Koch et al. 2021). If we consider a heat tracer test where we create a breakthrough curve like those in Fig. 3, we can model the behavior by

84 $$\begin{aligned} \frac{\partial T_{\mathrm{f}}^{*}}{\partial t^{*}} + \langle u_{z} \rangle \frac{\partial T_{\mathrm{f}}^{*}}{\partial z^{*}} - D_{\mathrm{f}}\frac{\partial ^{2} T_{\mathrm{f}}^{*}}{\partial z^{*2}} = \frac{{\overline{D}} A}{ V_{\mathrm{f}}} \frac{ (T_{\mathrm{s}}^{*} - T_{\mathrm{f}}^{*})}{L^{*}}, \end{aligned}$$

where ${\overline{D}}$ is a certain average of thermal diffusivity of the porous media, and $L^{*}$ is a characteristic length that defines the heat flux between the solid–fluid boundary. The formulation is similar to our study of reactive transport with first-order kinetics. Therefore, it is possible to apply the same techniques described in this work to obtain the “reaction order” and infer pore-scale information.

## Conclusion

Mineral reaction kinetics defined at the pore scale are not necessarily valid at the Darcy scale. We utilize a bundle-of-tubes model to study the modeling of dissolution kinetics in porous media at the Darcy scale. By adding an exponent, *n* (i.e., the Darcy-scale reaction order), to first- and second-order kinetics, the resulting constitutive relation approximates the average dissolution rate of the bundle-of-tubes model under specific experimental conditions. Using the pore-size and the effective-surface-area ratio distributions to characterize the tube bundles, we expand the solute concentration of dissolving species with Taylor series and thus relate the Darcy-scale Damköhler number, ${\text{Da}}_{\mathrm{d}}$, and reaction order with the distribution moments. The Taylor series expansions show that the Darcy-scale reaction order of first-order kinetics is the inverse square root of Tucker’s congruence coefficient (also known as the cosine similarity) between the pore sizes and effective-surface-area ratios. Therefore, an increase of reaction order indicates an increase of pore-scale heterogeneity. Such a relation gives a mechanistic meaning to the reaction order.

Furthermore, we simulate flow-through experiments of dissolving porous media at the laboratory as well as the field scale and discuss how one can utilize the constitutive relation by fitting a solute concentration breakthrough curve with ${\text{Da}}_{\mathrm{d}}$ and *n* as unknowns. As an additional benefit, we discuss cases of the flow-through experiments where the shape factor of longitudinal dispersivity is also considered as a fitting parameter. The inversion is successful, and the fitted parameters are close to the prescribed parameters calculated by the moments of pore-size and effective-surface-area ratio distributions. We infer that:

1. detailed pore-scale information (characterized by functions of moments) can be inferred using averaged Darcy-scale quantities (such as solute concentration), and
2. by analyzing the solute concentration of dissolving minerals over time using flow-through experiments, we can acquire the Darcy-scale reaction order and the dispersion coefficient, which represent heterogeneity at the pore scale.

The relations we derived provide us a quantitative approach to measure the spatial heterogeneity of a porous domain using the Darcy-scale reaction order and reveal a mechanistic explanation for $n>1$.

## Appendix 1: Derivation of the Longitudinal Dispersivity Using Spatial Moment Analysis

We introduce an advection equation of solute concentration in a pore

85 $$\begin{aligned} \frac{\partial C^{*}}{\partial t^{*}} + \langle u_{z} \rangle \frac{\partial C^{*}}{\partial z^{*}} = 0. \end{aligned}$$

The velocity, $\langle u_{z} \rangle$, is defined using Eq. (26)

86 $$\begin{aligned} \frac{\partial C^{*}}{\partial t^{*}} + \frac{r^{2}}{8} \frac{\Delta P}{\eta L_{z}}\frac{\partial C^{*}}{\partial z^{*}} = 0. \end{aligned}$$

When injecting a solute pulse at $z=0$, the center of mass of the solute is located at

87 $$\begin{aligned} z^{*}(t^{*}; r) = r^{2} \frac{\Delta P}{8 \eta L_{z}} t^{*}. \end{aligned}$$

Similar to the volume averaging procedure, Eq. (47), we define the center of mass in a bundle of tubes system

88 $$\begin{aligned} \bar{z}^{*}(t^{*}) = \frac{\displaystyle \int _{0}^{\infty } z^{*}(t^{*}; r) r^{2} p(r) {\mathrm{d}}r}{\displaystyle \int _{0}^{\infty } r^{2} p(r) {\mathrm{d}}r} = \frac{\mathrm{E}(r^{4})}{\mathrm{E}(r^{2})} \frac{\Delta P}{8 \eta L_{z}} t^{*}. \end{aligned}$$

Following the procedures of spatial moment analysis (Goltz and Roberts 1987; Dentz and de Barros 2013; Lee et al. 2018; Natarajan and Kumar 2018), the mean velocity is

89 $$\begin{aligned} \bar{u} = \frac{{\mathrm{d}} \bar{z}^{*}}{{\mathrm{d}}t^{*}} = \frac{\mathrm{E}(r^{4})}{\mathrm{E}(r^{2})} \frac{\Delta P}{8 \eta L_{z}} \end{aligned}$$

which is the volume averaged velocity, Eq. (32). The change of spatial variance over time is

90 $$\begin{aligned} \sigma _{z}^{2}&= \frac{\displaystyle \int _{0}^{\infty } (z^{*}(t^{*}; r))^{2} r^{2} p(r) {\mathrm{d}}r}{\displaystyle \int _{0}^{\infty } r^{2} p(r) {\mathrm{d}}r} - ({\bar{z}}^{*}(t^{*}))^{2} \end{aligned}$$

91 $$\begin{aligned}&= \left( \frac{\mathrm{E}(r^{6})}{\mathrm{E}(r^{2})} - \frac{\mathrm{E^{2}}(r^{4})}{\mathrm{E^{2}}(r^{2})} \right) \left( \frac{\Delta P}{8 \eta L_{z}} t^{*} \right) ^{2} \end{aligned}$$

92 $$\begin{aligned}&= \left( \frac{\mathrm{E}(r^{6}) \mathrm{E}(r^{2})}{\mathrm{E^{2}}(r^{4})} -1 \right) \left( \frac{\mathrm{E}(r^{4})}{\mathrm{E}(r^{2})} \frac{\Delta P}{8 \eta L_{z}} t^{*} \right) ^{2} \end{aligned}$$

93 $$\begin{aligned}&= \left( \frac{\mathrm{E}(r^{6}) \mathrm{E}(r^{2})}{\mathrm{E^{2}}(r^{4})} -1 \right) ({\bar{u}} t^{*})^{2} \end{aligned}$$

The longitudinal dispersion coefficient is

94 $$\begin{aligned} D_{\mathrm{L}} = \frac{1}{2} \frac{{\mathrm{d}} \sigma _{z}^{2}}{{\mathrm{d}} t} = \left( \frac{\mathrm{E}(r^{6}) \mathrm{E}(r^{2})}{\mathrm{E^{2}}(r^{4})} -1 \right) {\bar{u}}^{2} t^{*}, \end{aligned}$$

which concludes the derivation of Eq. (35). The nondimensional solute transport equation is

95 $$\begin{aligned} \frac{\partial {\overline{C}}}{\partial t} + {\text{Pe}}_{\mathrm{d}} \frac{\partial {\overline{C}} }{\partial z} - \left( 1 + ({\text{Pe}}_{\mathrm{d}})^{2} t\, f(r) \right) \frac{\partial ^{2} {\overline{C}}}{\partial z^{2}} = 0. \end{aligned}$$

Following Crank (1975), the fundamental solution of the aforementioned transport equation is

96 $$\begin{aligned} {\overline{C}}(z, t) = \frac{1}{\sqrt{4\pi (t + ({\text{Pe}}_{\mathrm{d}} t)^{2}/2)}} \exp {\left( -\frac{(z-z_{0}-{\text{Pe}}_{\mathrm{d}} t)^{2}}{4 (t+({\text{Pe}}_{\mathrm{d}} t)^{2}/2)}\right) }. \end{aligned}$$

We test the validity of the dispersion coefficient by comparing with the volume-averaged solute concentration. We consider two cases of pore size distributions $R_{1}$ and $R_{2}$ with initial injection at $z_{0}=0$ and ${\text{Pe}}_{\mathrm{d}}=10$.

Figure 8 shows the comparison between ${\overline{C}}$ and $C_{\mathrm{avg}}$ at different time steps. When the travel distance increases, the difference between ${\overline{C}}$ and $C_{\mathrm{avg}}$ increases. Such effect is more pronounced when $\sigma$ is larger, as shown in the right panel of Fig. 8. The higher-order method of moments is employed for better modeling of dispersion effects (Chatwin 1970; Zhang et al. 2008; Vikhansky and Ginzburg 2014; Jiang and Chen 2019). However, we limit our analysis to second order to focus on the topic of Darcy-scale reaction order.

## Appendix B Taylor Series of the Averaged Second-Order Kinetics Model

The averaged second-order kinetics model, Eq. (65), is

97 $$\begin{aligned} {\overline{C}}^{\prime } = {\text{Da}}_{\mathrm{d}} \left( 1-{\overline{C}}^{2}\right) ^n. \end{aligned}$$

We show derivations of expanding Eq. (97) using a Taylor series. A Taylor series expansion of ${\overline{C}}$ around $t=0$ is

98 $$\begin{aligned} {\overline{C}}(t) = {\overline{C}}(0) + {\overline{C}}^{\prime }(0)t + \frac{{\overline{C}}^{\prime \prime }(0)}{2} t^{2} + \frac{{\overline{C}}^{\prime \prime \prime }(0)}{6} t^{3} + O(t^{4}), \end{aligned}$$

which consists of derivatives of ${\overline{C}}$. Differentiate Eq. (97)

99 $$\begin{aligned} {\overline{C}}^{\prime \prime } = -2{\text{Da}}_{\mathrm{d}} n \left( 1-{\overline{C}}^{2}\right) ^{n-1} {\overline{C}} \,{\overline{C}}^{\prime }. \end{aligned}$$

The initial condition, ${\overline{C}}(0)=0$, leads to ${\overline{C}}^{\prime \prime }(0)=0$, which corresponds to the second-order term in Eq. (64). Instead of performing further differentiation, we rearrange Eq. (97)

100 $$\begin{aligned} {\overline{C}}^{'} = {\text{Da}}_{\mathrm{d}} \left( 1-{\overline{C}}\right) ^n \left( 1+{\overline{C}}\right) ^n. \end{aligned}$$

Applying the binomial approximation to $(1+{\overline{C}})^n$ yields

101 $$\begin{aligned} {\overline{C}}^{'} \approx {\text{Da}}_{\mathrm{d}} \left( 1-{\overline{C}}\right) ^n \left( 1+n{\overline{C}}\right) {\text{ for }} |n{\overline{C}} |\ll 1. \end{aligned}$$

Since we are interested in derivatives around $t=0$ and given the initial condition ${\overline{C}}(0)=0$, and since $n=O(1)$, the constraint of $|n{\overline{C}} |\ll 1$ is valid. Differentiate Eq. (101)

102 $$\begin{aligned} {\overline{C}}^{''} = {\text{Da}}_{\mathrm{d}} n {\overline{C}}^{'} \left( -\left( 1-{\overline{C}}\right) ^{n-1} \left( 1+n{\overline{C}}\right) + \left( 1-{\overline{C}}\right) ^n \right) , \end{aligned}$$

which retains the property ${\overline{C}}^{''}(0)=0$. Further differentiate and omit ${\overline{C}}^{''}$

103 $$\begin{aligned} {\overline{C}}^{'''}&= {\text{Da}}_{\mathrm{d}} n {\overline{C}}^{'} \Big ((n-1)\left( 1-{\overline{C}} \right) ^{n-2}\left( 1+n{\overline{C}}\right) {\overline{C}}^{'} \end{aligned}$$

104 $$\begin{aligned}&\quad -n \left( 1-{\overline{C}}\right) ^{n-1} {\overline{C}}^{'} - n\left( 1-{\overline{C}}\right) ^{n-1} {\overline{C}}^{'} \Big ). \end{aligned}$$

Hence,

105 $$\begin{aligned} {\overline{C}}^{'''}(0) = -({\text{Da}}_{\mathrm{d}})^{3}\,n(n+1). \end{aligned}$$

Therefore, the Taylor series with an approximated third-order derivative is

106 $$\begin{aligned} {\overline{C}}(t) = {\text{Da}}_{\mathrm{d}} t - \frac{n(n+1)}{6}({\text{Da}}_{\mathrm{d}}t)^{3} + O(t^{3}). \end{aligned}$$
